# Resonance assignment of the Shank1 PDZ domain

**DOI:** 10.1007/s12104-022-10069-4

**Published:** 2022-01-27

**Authors:** Anna Sánta, András Czajlik, Gyula Batta, Bálint Péterfia, Zoltán Gáspári

**Affiliations:** 1 Faculty of Information Technology and Bionics, Pázmány Péter Catholic University, Práter u. 50/A, 1083 Budapest, Hungary; 2 Faculty of Science and Technology, Institute of Chemistry, Department of Organic Chemistry, University of Debrecen, Egyetem tér 1, 4032 Debrecen, Hungary

**Keywords:** Shank1, PDZ domain, Helical propensity, Monomeric

## Abstract

Shank proteins are among the most abundant and well-studied postsynaptic scaffold proteins. Their PDZ domain has unique characteristics as one of its loop regions flanking the ligand-binding site is uniquely long and has also been implicated in the formation of PDZ dimers. Here we report the initial characterization of the Shank1 PDZ domain by solution NMR spectroscopy. The assigned chemical shifts are largely consistent with the common features of PDZ domains in general and the available Shank PDZ crystal structures in particular. Our analysis suggests that under the conditions investigated, the domain is monomeric and the unique loop harbors a short helical segment, observed in only one of the known X-ray structures so far. Our work stresses the importance of solution-state investigations to fully decipher the functional relevance of the structural and dynamical features unique to Shank PDZ domains.

**Supplementary Information:**

The online version contains supplementary material available at 10.1007/s12104-022-10069-4.

## Biological context

Members of the Shank (SH3 and ankyrin repeat containing protein) family are among the most abundant members of postsynaptic scaffold proteins (Kursula 2019). Mutations in Shank proteins have been linked to a number of neurological disorders (Sala et al. 2015). Shank proteins are modular proteins consisting of several globular domains, an SPN, an SH3, a PDZ and a SAM domain, an ankyrin repeat region (ARR), as well as a number of non-structured regions including a proline-rich segment. Shank proteins can establish a large number of interactions with a multitude of proteins as listed in the recently launched PSINDB database (https://psindb.itk.ppke.hu). Recently, the role of autism-related mutations in altering the preferred interdomain orientations was suggested, with the SH3 and PDZ domains forming different contacts with the ARR and the subsequent linker region (Bucher et al. 2021).

PDZ domains in general have been studied intensively in the past decades. The role of the loops flanking the peptide binding site have been extensively discussed in the literature as they provide additional modulation to the partner binding properties by mediating allosteric effects (Mostarda et al. 2012; Murciano-Calles et al. 2014; Kumawat and Chakrabarty 2017; Kovács et al. 2020). Comparative studies of the structure and dynamics of PDZ domains can reveal novel aspects of the common and unique features of PDZ domains, not directly available from the study of some common examples like the PSD-95 PDZ3 (Dudola et al. 2020). Shank PDZ domains contain an unusually long β2-β3 loop (Supplementary Fig. S1.) that is highly conserved through all three isoforms and species, therefore might mediate inter- and intramolecular interactions unique to these proteins. (Im et al. 2003) Although there are a number of Shank PDZ domains available in the PDB, all of them were determined by X-ray crystallography. There is even some controversy about the monomeric or dimeric nature of Shank PDZ domains based on these structures. Shank1 PDZ was first described as a dimer by Im et al. (Im et al. 2003), followed by a completely different dimer conformation of Shank3 PDZ observed by Zeng et al. (Zeng et al. 2016), but more recent crystallization experiments of Shank3 PDZ do not suggest dimerization (Ponna et al. 2018). Shank PDZ domains are also remarkably promiscuous but show a high preference for main interaction partner GKAP (DLGAP1, Disks large-associated protein 1), with a possibility of different conformations being responsible for binding selectivity (Zeng et al. 2016). Solution-state dynamics and partner binding studies by NMR are expected to contribute significantly to our detailed understanding of structure-dynamics-function relationships of the Shank PDZ domains and to establish mechanistic details of possible allosteric effects. To our knowledge, there are currently no published solution-state NMR studies on Shank PDZ domains.

Here we report the expression, purification and resonance assignment of the rat Shank1 PDZ domain construct. Analysis of R_1_ and R_2_ relaxation parameters suggests that the domain is monomeric in solution.

## Methods and experiments

### Cloning, protein expression and purification

Our Shank1 PDZ construct spans from G654 to K768 on the rat Shank1 reference sequence (UniProt ID:Q9WV48) which in this region matches exactly—both the positions and the sequence—that of the human protein (UniProt ID:Q9Y566). The expressed construct has N terminal 6xHis tag. Briefly, the insert was amplified from a plasmid bearing the full length rat Shank-1 ORF (kindly provided by Enora Moutin), with 5′-tttttcatatgGGGAGTGATTACATCATCAAGGAG forward and 5′-tttttggatcctcaCTTGTGGACAGCCTCGT reverse primers. Using NdeI and BamHI sites, the gel extracted PCR product was ligated into a modified pET-15b vector (Novagen) that contains a tobacco etch virus (TEV) protease cleavage site instead of the thrombin sequence. After verifying the clones by Sanger sequencing, constructs were transformed into BL21 (DE3) cells (Novagen). Protein production was induced with 1 mM IPTG (Sigma) at 4 MFU cell density and then cells were shaken at 20 °C for 16 h for expression. Cells were grown in LB medium for unlabeled protein production. For isotopically labeled protein production freshly prepared M9 medium was used (22 mM KH_2_PO_4_; 50 mM Na_2_HPO_4_·2H_2_O; 8.5 mM NaCl; 2 mM MgSO_4_; 0.1X vitamine mix; 0.2X Trace metal mix, (Studier 2005) supplemented with 0.4% [^13^C]-D-glucose or unlabeled Glucose and 0.25% ^15^NH_4_Cl (Cambridge Isotope Laboratories, Cambridge, MA). After harvesting, cells were extracted by ultrasonic homogenization in 10% suspension using a lysis buffer (50 mM NaPi, 300 mM NaCl, pH 7.4). His-tagged proteins were purified on Bio-Scale™ Mini Nuvia™ IMAC Ni-affinity column (Bio-Rad), followed by His-tag cleavage with TEV protease. This protocol results in a 4-residue tag (GSHM) attached to the N-terminus of the wild-type sequence. The recombinant protein was further purified by ion exchange chromatography, using Bio-Scale™ Mini Macro-Prep® High Q column. In this case, the Shank protein was collected in the flow through fraction. Samples were concentrated to 1.0 mL and applied to size exclusion chromatography before NMR experiments using ENrich™ SEC 70 10 × 300 mm column (Bio-Rad). The recombinant protein was eluted in a low salt NaPi Buffer (50 mM NaPi; 20 mM NaCl, 0.02% NaN_3_; pH 7.4). Analysis of the purity and exact molecular weight of the protein was performed by SDS-PAGE and LC–MS, respectively, and the structural integrity of the construct was assessed with ECD, while ligand-binding capability was tested by a simple IMAC (Bio-Rad Profinity™ IMAC Resin) pull-down assay using the C-terminal segment of the GKAP protein as the bait. The GKAP C-terminal construct spans the last 186 residues of *Rattus norvegicus* DLGAP1 (Uniprot P97836), which is 100% identical to the same segment in the human protein. Purification protocol for this construct was largely identical to those described above for Shank PDZ except for the use of a Bio-Scale™ Mini Macro-Prep® High S column in place of the Q column and that the protein was collected in the elution (50 mM NaPi; 20 mM NaCl, pH 7.0).

### NMR spectroscopy and data analysis

500 µl samples containing 50 mM NaPi, 20 mM NaCl, 0.02% NaN_3_ at pH 7.4 with 8% D_2_O and 200 ﻿µM protein were measured on a Bruker Avance NEO 700 MHz spectrometer at 298 K. After collecting ^15^N-^1^H HSQC spectra, triple-resonance HNCO, HN(CA)CO, HNCA, HN(CO)CA, HNCACB and HN(CO)CACB experiments were performed for assignment of labelled backbone nuclei. ^15^N-^1^H-TOCSY, ^15^N-^1^H-NOESY, 3D (H)CCH-TOCSYali, 2D ^1^H-’H NOESY, HBCBCGCDHD as well as HBCBCGCDCEHE spectra were also recorded to aid sidechain assignment. NMR spectra were processed in Bruker TopSpin, and analysis was performed in CARA (Keller 2004) and CCPNMR 2.5.2 (Vranken et al. 2005). Secondary structure estimation based on chemical shifts was performed with TALOS-N (Shen and Bax 2013).

For ^15^N R_1_ and R_2_ relaxation measurements (Farrow et al. 1994), pseudo-3D spectra were recorded and processed with TopSpin to extract the individual 2D spectra corresponding to the different delay times. Peak analysis and relaxation curve fitting was performed with NMRFAM-Sparky (Lee et al. 2015) using the ‘rh’ command. Relaxation rate errors were adjusted to 7% of the values where the initial estimate was lower than this as suggested by Song and Markley (Song and Markley 2003). After excluding data with ^15^N R_2_/R_1_ ratios diverging from the average with more than ± 1 standard deviation, the parameters were analyzed with Tensor2 (Dosset et al. 2000). The necessary peak list format conversions between the programs and the initial analysis of ^15^N R_2_/R_1_ ratios were performed with in-house Perl scripts. Structure-based estimation of the rotational correlation time was performed with HydroPro (Ortega et al. 2011).

Structural models of the domain, used for chemical shift estimation and in the Tensor2 analysis, corresponding to all residues in our construct were generated with MODELLER (Webb and Sali 2016) using the template structures 1q3o and 7a9b. Secondary structure elements were assigned using DSSPcont (Andersen et al. 2002).

Chemical shifts estimated from the structural model were calculated using SHIFTX (Neal et al. 2003) along with sequence-dependent random coil shifts reported by Tamiola et al*.* (Tamiola et al. 2010) as implemented in CoNSEnsX^+^ (Dudola et al. 2017).

## Extent of assignments and data deposition

### The Shank1 PDZ domain is functional and folded

**Fig. 1:**
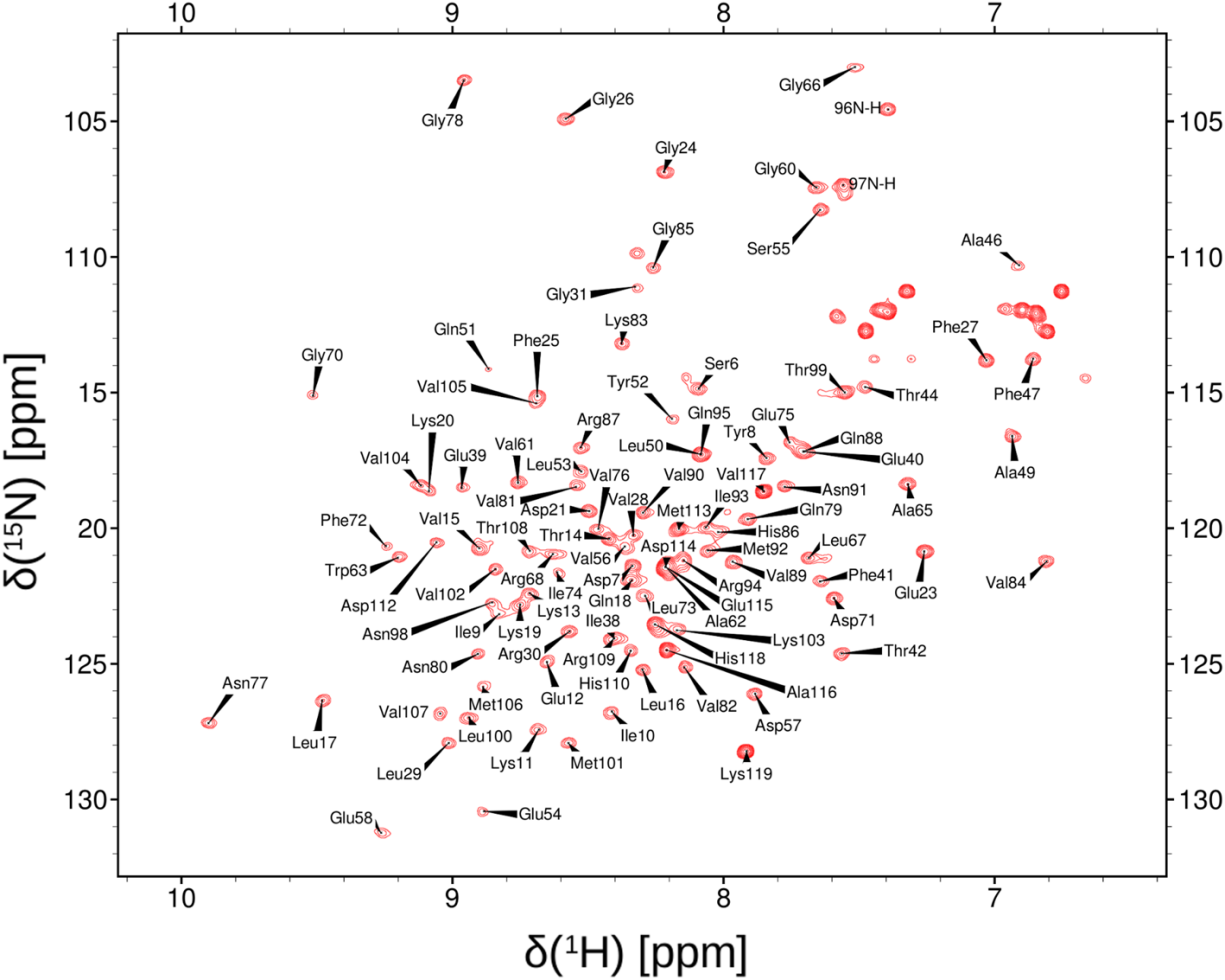
^1^H-^15^N HSQC spectrum of the Shank1 PDZ domain. The majority of the assigned backbone amide peaks are labeled (except for residues in the expression tag and ambiguous residues in the β2-β3 loop. Residue numbering corresponds to that of the construct. Figure prepared with NMRFAM-Sparky (Lee et al. 2015)

The Shank1 PDZ domain was successfully expressed and ECD analysis in 50 mM NaPi, 20 mM NaCl at pH 7.4 is consistent with an intact globular structure dominated by β-sheets (Supplementary Fig. S2). The ^1^H-^15^N HSQC spectrum of the construct indicates a well-folded structure with high signal dispersion and generally well-resolved peaks (Fig. 1). The pull-down assay proved that the expressed PDZ domain is functional as it can bind to the C-terminal segment of rat GKAP. (Supplementary Fig. S3). More details of this interaction will be described elsewhere.

### Completeness of the assignment

Below, residue numbering refers to the positions in our PDZ construct and the positions corresponding to the full Shank1 sequence are given in parentheses. Backbone resonances of residues in the range 5–119 (G654-K768) could be established with the exception of residues 32–35 (A681-Q684) of the β2-β3 loop. Here, the alanines at positions 32 (A681) and 34 (A683) could be tentatively assigned but their exact identity is ambiguous. The N-terminal region corresponding to the 4 residues from the expression vector, could only partially be assigned probably due to the mobility of the region. In addition, the amide nitrogen chemical shifts for the non-proline residues 5 (G654), 22 (S671), 36 (T685), 59 (G708) and 69 (M718), otherwise assigned with high confidence, could not be unambiguously identified. The latter four are all solvent-exposed amides located in loop regions. Side-chain assignments could not be completed for several proline as well as for lysine and arginine residues, most notable is the arginine at position 64 (R713), for which only the backbone resonances could be identified. This residue is located in the first of the α-helices characteristic of PDZ structures, adjacent to the β1–β2 loop at the carboxylate binding region of the ligand binding site.

Overall, 115 of the 119 C_α_ resonances were identified, and 101 of the 113 amide groups (when accounting for the N-terminus and prolines) could be assigned. Of the side-chain nitrogen resonances, the indole nitrogen of the single Trp in the construct (W712), all four asparagine amides as well as two of the six glutamine amides could be identified. Regarding all possible proton resonances, our assignment is approximately 68% complete. Detailed statistics on the assigned shifts by atom type are shown in Supplementary Table S1.

The anomalous ^13^C carbonyl chemical shifts of Thr42 (691) and ^15^N amide shift of Ala46 (695) might be due to the aromatic cluster near the interface of the long β2-β3 loop and the backside of the ligand-binding cleft, although the orientations of the side chains observed in the X-ray structures do not allow unambiguous explanation of the observed deviations (Supplementary Fig. S4).

### Secondary structure and comparison with known structures

In a sequence-based search with 50% identity cutoff, 18 entries can be found in the PDB, all determined by X-ray, corresponding to Shank1 and Shank3 PDZ structures. These correspond to a total of 44 coordinate sets when accounting for multiple conformations in the entries. Generally, the β2-β3 loop region is unresolved in most of the available X-ray structures and even where the coordinates are available, the B-factors are uniformly high compared to the rest of the structure.

TALOS-N predicts five β-strand regions, corresponding to Tyr8-Lys19, Phe27-Arg30, Gln51-Asp57, Phe72-Gln79 and Thr99-Thr108, as well as and three helical segments, Ile38 to Glu40, from Val61 to Ala65 and from His86 to Gln95. The strands correspond well to those assigned by DSSP for the structural models (see below) with only a small interruption in strand 4 (Fig. S4), also consistent with the TALOS-N prediction. The first of the helical regions lies at the N-terminal region of the long β2-β3 loop, whereas the second and the third correspond to the helices common to the general PDZ fold. Visual inspection and DSSP analysis revealed that only one of the available Shank PDZ structures, corresponding to chain “A” of the entry 7a9b (Ali et al. 2021) exhibits a 3_10_-helix (DSSP state “G”) in the region 36–40. The two-residue discrepancy is also reflected in the calculated high secondary CA shifts of Thr36, not matched by the experimental value (see below). These observations suggest that this helix is indeed at least partially formed in solution but is shorter than in the structure 7a9b.

**Fig. 2:**
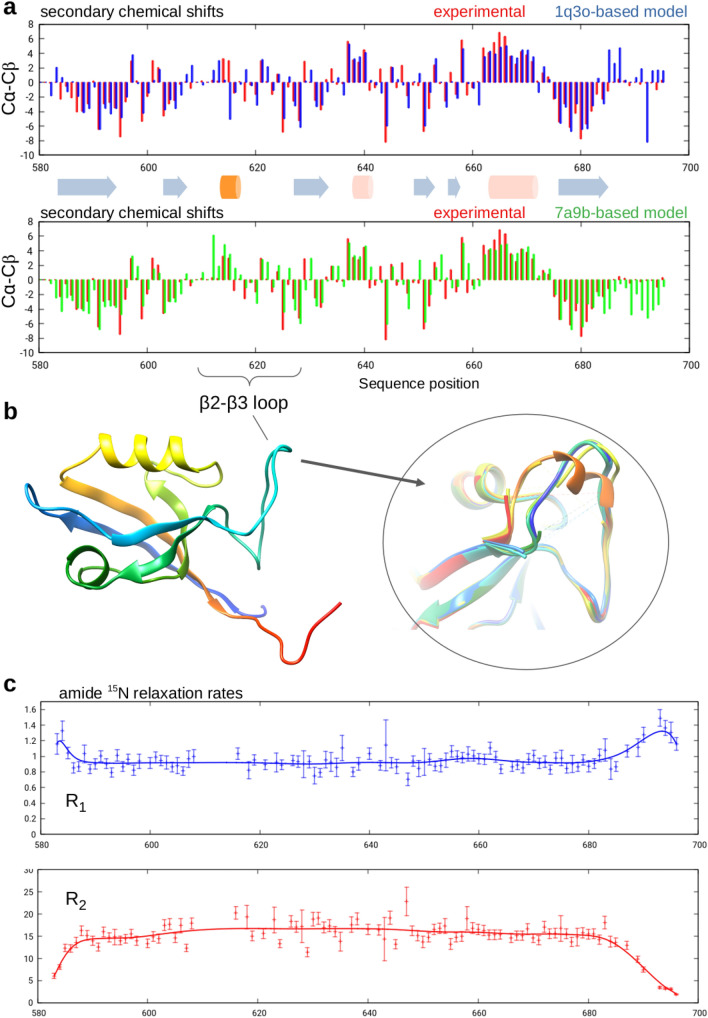
Experimental (red) and calculated (blue: 1q3o-based model, green: 7a9b-based model) difference of the ^13^C_ɑ_-^13^C_β_ secondary chemical shifts for the Shank1 PDZ domain (a). Secondary structure annotation was derived by DSSPcont (Andersen et al. 2002) using the models. The orange helix is predicted by TALOS and is only present in the 7a9b structure. For the preparation of the figure, the chemical shifts of the ambiguously assigned Ala32 and Ala34 of the β2-β3 loop were also included. Ribbon representation of the structure of the Shank1 domain drawn from the structure 1q3o (b). The circled inset shows the β2-β3 loop in 44 Shank1/Shank3 PDZ conformations in all chains from 18 deposited PDB structures as superimposed with Chimera (Pettersen et al. 2004). Note the unresolved residues in the majority of structures and the presence of a helical segment in only one of them. Amide ^15^N R_1_ and R_2_ relaxation rates (c). The smoothed bezier curves are shown to highlight the general trends

We have prepared structural models spanning all residues in our construct based on the PDB entries 1q3o (Im et al. 2003) and 7a9b, where the β2-β3 loop is resolved. We compared the C_ɑ_ and C_β_ secondary chemical shifts to those estimated from these models using SHIFTX and sequence-dependent random coil values (Fig. 2) as integrated into the CoNSEnsX^+^ server (Dudola et al. 2017). These show reasonably good agreement throughout the sequence, justifying the validity of our backbone assignment. The largest discrepancies are observed for the β2-β3 loop region with the 7a9b-based model exhibiting slightly better agreement because of the presence of the helical segment.

### Relaxation analysis

^15^N R_1_ and R_2_ relaxation rates could be extracted for 93 residues. The assigned but overlapping peaks of residues 62/114/115, 40/88 and 38/109 were omitted from rate fitting. Based on the analysis of the average ^15^N R_2_/R_1_ ratios, a total 16 residues, including the flexible C-terminal tail, were excluded from the estimation of the overall rotation correlation time (Supplementary Table S2). The τ_c_ value estimated by Tensor2 is 10.66 ns, in reasonable agreement with the range of harmonic mean correlation times of 10.5–13.2 ns obtained from the different structural models with HydroPro. According to Daragan’s formula (Daragan and Mayo, 1997), the global correlation time of folded proteins at a given temperature can also be predicted from sequence length. In our case, a length of 119 residues at T = 298 K provides an estimation of 8.7 ns for the monomer and 16.6 ns for a dimer. This strongly suggests that the isolated Shank1 PDZ domain is monomeric in solution.

## Summary

We have successfully expressed the PDZ domain of the Shank1 protein and performed NMR measurements for resonance assignment and estimation of the rotational correlation time. To our knowledge, this is the first NMR study of the otherwise intensively investigated Shank1 PDZ domain. Our results clearly show that the domain has a well-folded globular structure with secondary structure distribution matching that expected from a characteristic PDZ domain fold. The encountered difficulties in achieving a full backbone amide and a more complete side-chain assignment are, at least in part, most likely due to the enhanced flexibility of some of the loop regions. We note here that for the closely related Shank3 PDZ domain, the conditions for which highest stability was observed included high salt concentrations and pH 8 (Ponna et al. 2018). Therefore, it is possible that under the conditions of our NMR investigations, with low salt and slightly lower pH, the stability of the domain is suboptimal.

Analysis of ^15^N R_1_ and R_2_ relaxation rates resulted in a τ_c_ that strongly suggests that under these conditions the Shank1 PDZ domain is monomeric. In contrast, the unliganded 1q3o crystal structure, determined at pH 4.5 is a dimer and its authors also observed that their construct is dimeric in solution (Im et al. 2012). The other dimeric structure reported forms a domain-swapped dimer where the N-terminal extension of the bound GKAP peptide is involved in contacts with the strands exchanged between the Shank PDZ monomers (Zeng et al. 2016). These observations might indicate the presence of a monomer–dimer equilibrium sensitive to the exact conditions used and the availability of binding partners.

The conformation and role of the uniquely long β2-β3 loop in Shank PDZ domains is also worth investigating. Our analysis of secondary chemical shifts shows that a short segment in this loop has helical propensity, but a short helix is only observed in one of the available X-ray structures in this region. We also note that in the recently published AlphaFold (Jumper et al. 2021) model for rat Shank1 (https://alphafold.ebi.ac.uk/entry/Q9WV48) this short segment is not modeled as helical, probably due to the absence of multiple templates with this feature.

Our initial analysis shows that solution-state NMR investigation of Shank PDZ domains can still provide valuable information about these molecules. We hope that our results pave the way for further detailed studies of the structure, dynamics and ligand-binding properties of Shank PDZ domains and contribute to the understanding of the diversity of PDZ structures in general.

## Supplementary Information

Below is the link to the electronic supplementary material.

Supplementary file1 (PDF 1051 KB)

## Data Availability

The assigned chemical shifts have been deposited in the BMRB under accession number 51126.
