# Supplementary material for: Resonance assignment of the Shank1 PDZ domain
Source: Biomol NMR Assign. 2022 Jan 27;16(1):121–7. doi: 10.1007/s12104-022-10069-4 (PMC9068651; doi:10.1007/s12104-022-10069-4)
Supplement: Supplementary file 1 — Supplementary file1 (PDF 1051 KB) [file 12104_2022_10069_MOESM1_ESM.pdf]

Supplementary material for

Resonance assignment of the Shank1 PDZ domain

Anna Sánta, András Czajlik, Gyula Batta, Bálint Péterfia, Zoltán Gáspári

Supplementary tables

Table S1

Completeness of the resonance assignment by atom type

**Atom type**	**Number of atoms in construct[1]**	**Number of assigned atoms**	**Percentage of assigned atoms**
Amide ^{1}HN (backbone)	113	101	89.4
Hα	132	121	91.7
Amide ^{15}N (backbone)	118	101	85.6
Carbonyl ^{13}C’ (backbone)	119	108	90.1
^{13}Cα	119	115	96.6
^{13}Cβ	106	99	93.4
All other side-chain ^{13}C (without Cβ)	239	96	40.2[2]
All non-exchangeable side-chain ^{1}H (including Hβ)[3]	470	312	66.4

[1]The number of the different atom types is taken from an empty table file generated by BMRB based on the sequence of the construct
[2]The relatively low percentage is partly due the lack of aromatic assignments
[3]Methyl protons are not counted separately, only as groups

Table S2

Shank1 PDZ residues with above and below-average T1/T2 (=R2/R1) amide N relaxation values that were excluded from the estimation of the τ_c with Tensor2.

**Residue number**	**Position in full Shank1 sequence**	**Residue name**	**R1 (1/s)**	**R2 (1/s)**	**R2/R1**	
6	655	Ser	1.158	6.050	5.225	τ_e
7	656	Asp	1.326	8.091	6.102	τ_e
29	678	Leu	0.812	17.419	21.452	R_{ex}
41	690	Phe	0.823	19.342	23.502	R_{ex}
53	702	Leu	0.751	18.829	25.072	R_{ex}
54	703	Glu	0.790	19.095	24.171	R_{ex}
60	709	Gly	0.797	17.630	22.120	R_{ex}
61	710	Val	0.902	18.925	20.981	R_{ex}
67	716	Leu	0.915	19.153	20.932	R_{ex}
70	719	Gly	0.703	22.852	32.506	R_{ex}
112	761	Asp	1.111	9.862	8.877	τ_e
113	762	Met	1.275	7.502	5.884	τ_e
116	765	Ala	1.491	3.440	2.307	τ_e
117	766	Val	1.360	3.237	2.380	τ_e
118	767	His	1.305	3.100	2.375	τ_e
119	768	Lys	1.156	1.931	1.670	τ_e

Supplementary figures

```
                             cov     pid  1 [        .         .         .         .         :         . 60
 1 SHANK1|663-757 100.0% 100.0%     TVLLQKK-DSEGFGFVLRGAKAQTPIEEFTPTPAFPALQYLESVDEGGVAWRAG-LRMGD
 2 SHANK3|570-664 100.0%  81.1%     VAILQKR-DHEGFGFVLRGAKAETPIEEFTPTPAFPALQYLESVDVEGVAWKAG-LRTGD
 3 PSD95|65-151     90.5%  22.1%     EITLERG--NSGLGFSIAGGTDNPHIGDDPSI-------FITKIIPGGAAAQDGRLRVND
 4 PSD95|160-246    90.5%  24.2%     EIKLIKG--PKGLGFSIAGGVGNQHIPGDNSI-------YVTKIIEGGAAHKDGRLQIGD
 5 PSD95|313-393    84.2%  21.1%     RIVIHRG--STGLGFNIVGGED------GEGI-------FISFILAGGPADLSGELRKGD
 6 GRIP1|53-136     87.4%  21.9%     VVELMKK-EGTTLGLTVSGGIDKD-----GKP-------RVSNLRQGGIAARSDQLDVGD
 7 GRIP1|150-238    91.6%  23.7%     TVEVTLHKEGNTFGFVIRGGAHDDRN-KSRPV-------VITCVRPGGPADREGTIKPGD
 8 GRIP1|252-336    88.4%  11.5%     LVEVAKT-PGASLGVALTTSVCCN----KQVI-------VIDKIKSASIADRCGALHVGD
 9 GRIP1|471-560    91.6%  18.4%     EVVLTAD-PVTGFGIQLQGSVFATETLSSPP--------LISYIEADSPAERCGVLQIGD
10 GRIP1|572-657    89.5%  20.8%     HVKLPKK-HSVELGITISSPSSRKP---GDPL-------VISDIKKGSVAHRTGTLELGD
11 GRIP1|672-754    86.3%  21.9%     TVELKR--YGGPLGITISG--TEEP---FDPI-------IISSLTKGGLAERTGAIHIGD
12 GRIP1|988-1070   86.3%  21.9%     KVTLYKDSGMEDFGFSVADGLLEKGV-------------YVKNIRPAGPGDLGG-LKPYD
   consensus/100%                     .h.l.h......hGh.l.s....................hlp.l..tu.u..ts.lp..D
   consensus/90%                      .l.l.tt...tshGhsltss..t................hlp.l..su.A.hsG.lp.sD
   consensus/80%                      .l.l.+t..stshGhsltuu.tpp......s........hlppl..uu.AtpsG.LchGD
   consensus/70%                      pl.L.+t..spshGhsltGuhspp.....tsh.......hlsplp.uGsAt+sG.LchGD

                             cov     pid 61          .         .         .        ] 99
 1 SHANK1|663-757 100.0% 100.0%     FLIEVNGQNVVKVGHRQVVNMIRQG--GNTLMVKVVMVT
 2 SHANK3|570-664 100.0%  81.1%     FLIEVNGVNVVKVGHKQVVGLIRQG--GNRLVMKVVSVT
 3 PSD95|65-151     90.5%  22.1%     SILFVNEVDVREVTHSAAVEALKEA--GSIVRLYVMRR-
 4 PSD95|160-246    90.5%  24.2%     KILAVNSVGLEDVMHEDAVAALKNT--YDVVYLKVAKP-
 5 PSD95|313-393    84.2%  21.1%     QILSVNGVDLRNASHEQAAIALKNA--GQTVTIIAQYK-
 6 GRIP1|53-136     87.4%  21.9%     YIKAVNGINLAKFRHDEIISLLKNV--GERVVLEVEYEL
 7 GRIP1|150-238    91.6%  23.7%     RLLSVDGIRLLGTTHAEAMSILKQC--GQEATLLIEYDV
 8 GRIP1|252-336    88.4%  11.5%     HILSIDGTSMEYCTLAEATQFLANT--TDQVKLEILPHH
 9 GRIP1|471-560    91.6%  18.4%     RVMAINGIPTEDSTFEEANQLLRDSSITSKVTLEIEFDV
10 GRIP1|572-657    89.5%  20.8%     KLLAIDNIRLDSCSMEDAVQILQQC--EDLVKLKIRKDE
11 GRIP1|672-754    86.3%  21.9%     RILAINSSSLKGKPLSEAIHLLQMA--GETVTLKIKKQT
12 GRIP1|988-1070   86.3%  21.9%     RLLQVNHVRTRDFDCCLVVPLIAES--GNKLDLVISRNP
   consensus/100%                     .lh.lst..h....ht.h..hlt.s...p.h.h.h....
   consensus/90%                      .lhtlst.th.th.httsh.hltps..tp.lhl.l....
   consensus/80%                      hlltlsshph.thshppshthlpps..sphlhl.l.h..
   consensus/70%                      plltlNulsltpsshppssthLpps..spplhLhl.hp.
```

Figure S1. Sequence alignment of the core region of the PDZ domains of selected human postsynaptic proteins. Figure prepared with MView (https://www.ebi.ac.uk/Tools/msa/mview/).

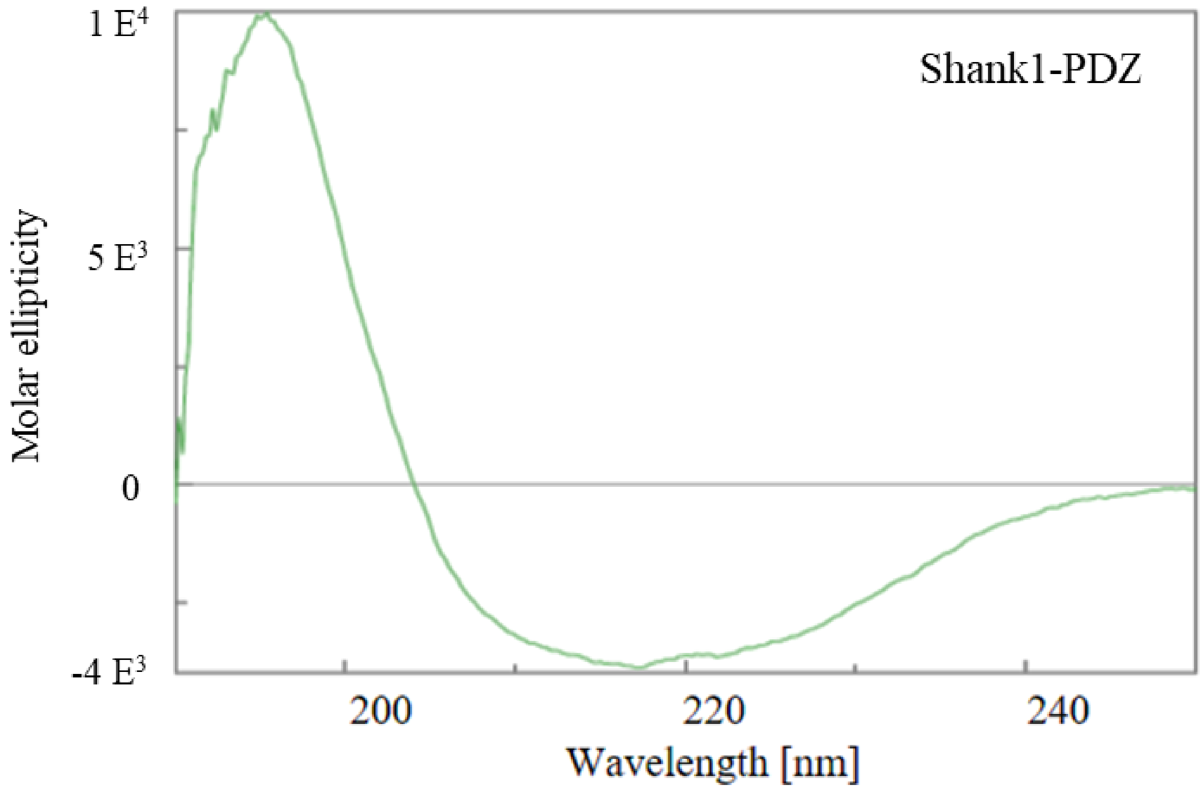

Figure S2. ECD spectrum of the Shank1 PDZ domain. The spectrum indicates a well-folded structure dominated by β-sheet structure. The buffer used was identical to that used for NMR analysis (50mM NaPi, 20mM NaCl, pH 7.4).

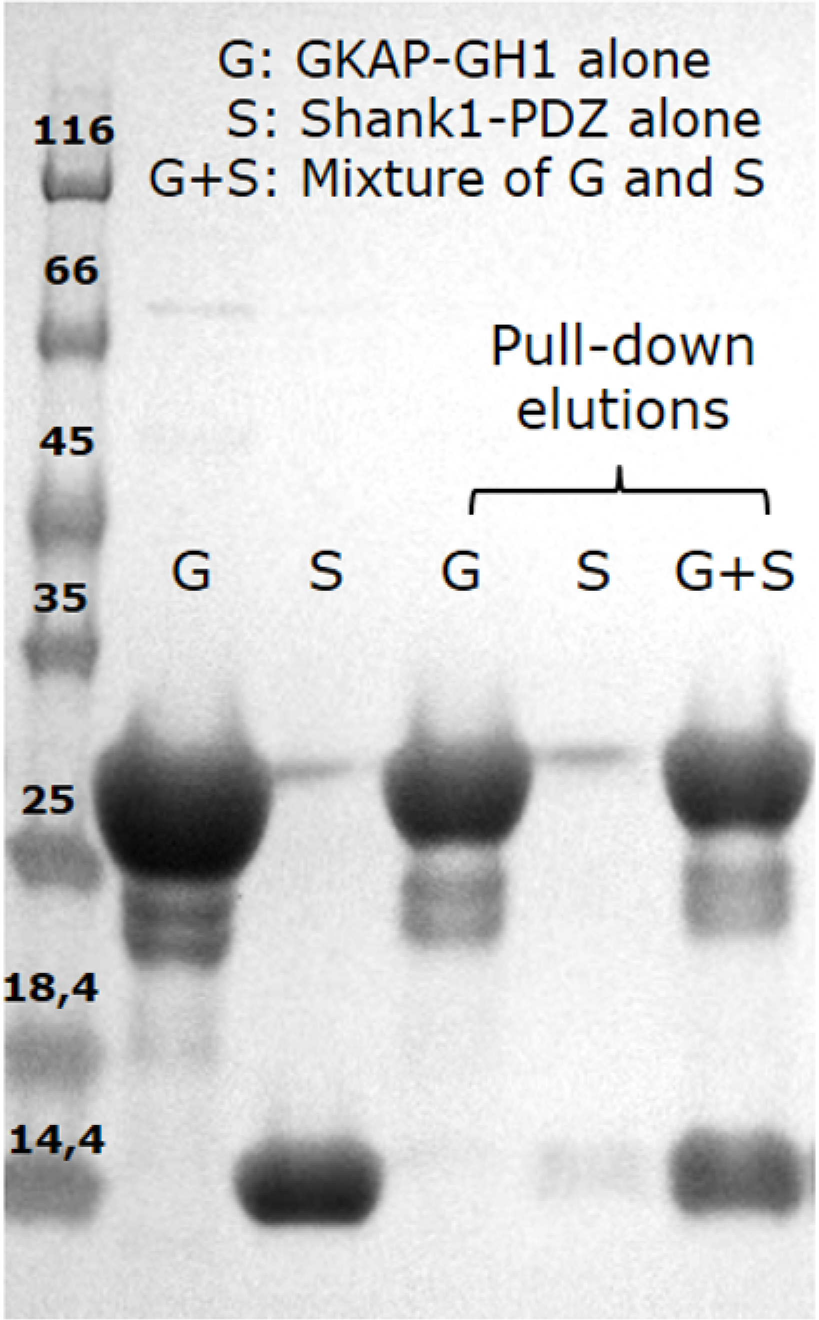

Figure S3. Pull-down experiment with Shank1 PDZ and the C-terminal region of GKAP. The pull.down affinity tag was attached to the Shank1 PDZ domain. The presence of the two bands corresponding to the MW of the partners in the G+S pull-down lane indicates the binding between Shank1 PDZ and the C-terminal segment of GKAP.

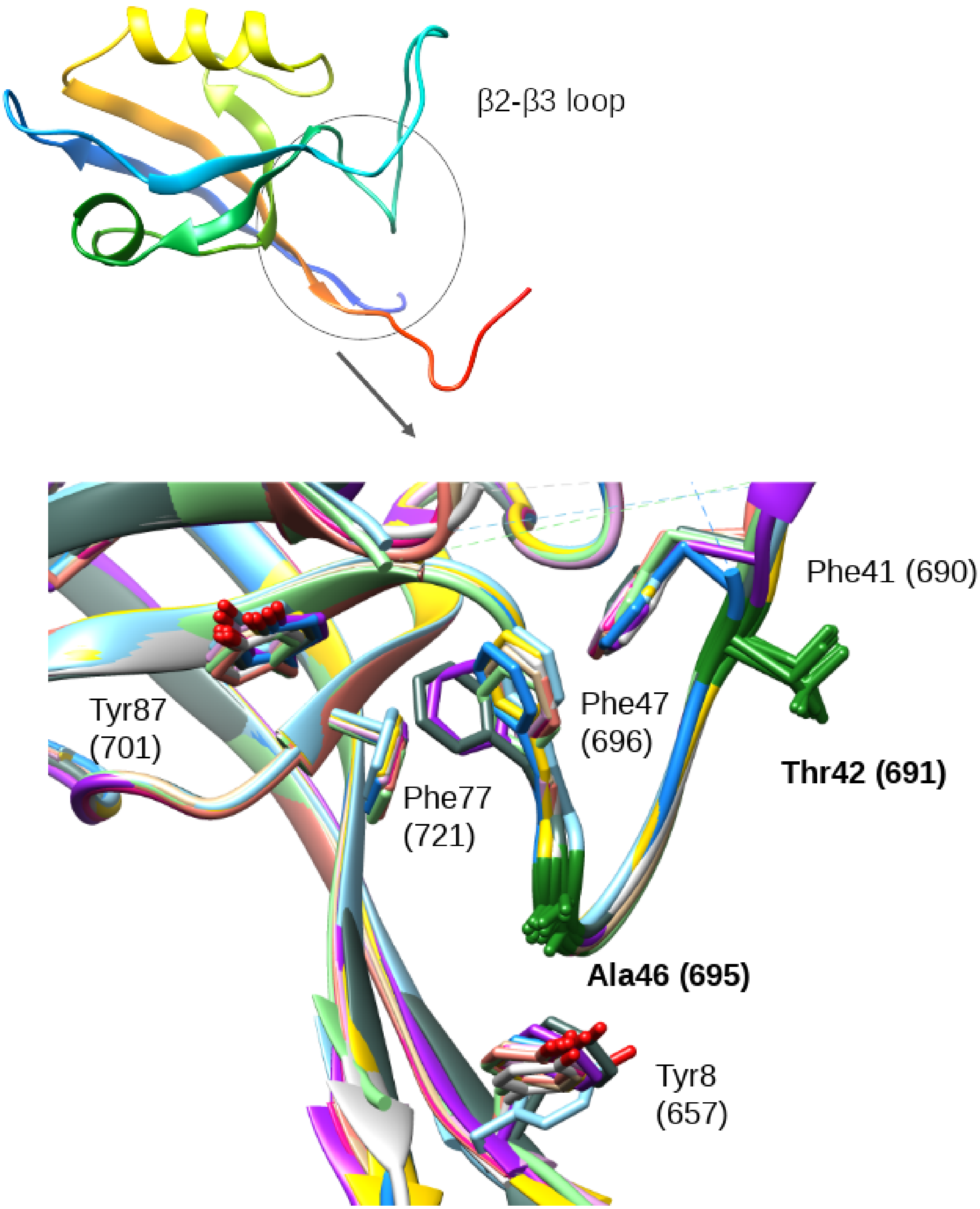

Figure S4. The aromatic cluster near the interface of the β2-β3 loop and the “backside” of the ligand binding cleft as a possible explanation for the unusual ^{13}C carbonyl and ^{15}N amide chemical shifts of Thr42 and Ala46, respectively (shown in dark green). The structures of all 44 Shank1/3 PDZ domains are shown in different colors to account for the (limited) side-chain conformational variability of the aromatic residues. Figure prepared with Chimera (Pettersen et al. 2004).
